# Carcinoma Cuniculatum of the Maxilla Arising From Oroantral Fistula: A Report of an Extremely Rare Case

**DOI:** 10.7759/cureus.37796

**Published:** 2023-04-18

**Authors:** Aya Muramatsu, Hiromasa Hasegawa, Kimihide Kusafuka, Makoto Suzuki

**Affiliations:** 1 Department of Pathology, Shizuoka General Hospital, Shizuoka, JPN; 2 Center for Clinical Pathology, Fujita Health University Hospital, Toyoake, JPN; 3 Hard Tissue Pathology Unit, Graduate School of Oral Medicine, Matsumoto Dental University, Shiojiri, JPN

**Keywords:** oral and maxillary pathology, diagnostic pathology, maxilla, odontogenic keratocyst, oroantral fistula, squamous cell carcinoma, carcinoma cuniculatum

## Abstract

Carcinoma cuniculatum (CC) is extremely rare in the maxilla. Here, we report a case of CC arising from an oroantral fistula (OAF). The patient was a 70-year-old Japanese man who was followed up for a non-closing OAF. Although there were no findings based on an intraoral examination, follow-up contrast-enhanced computed tomography and magnetic resonance imaging showed a 22-mm mass in the maxilla close to the OAF. Histologically, cystic and endophytic papillary proliferation of squamous epithelium with abundant keratinization mimicking rabbit burrows occupied the alveolar bone. This tumor was directly connected to the atypical proliferation of the covering epithelium of the OAF. The tumor cells showed mild cytological atypia and a few mitoses. Finally, the patient was diagnosed with CC arising from an OAF. CC is often misdiagnosed; nonetheless, the unique endophytic, branching, and tunnel-like structure is a hallmark of this tumor. We present the first well-documented case of CC arising from an OAF, discuss its diagnostic features, and highlight its differences from other common benign and malignant pathological entities.

## Introduction

Carcinoma cuniculatum (CC) is a rare subtype of well-differentiated squamous cell carcinoma (SCC), which was first described by Aird et al. in 1954 [1]. They initially referred to this neoplasm as epithelioma cuniculatum on the plantar surface of the foot based on its clinicopathological findings and biological behavior; later, several investigators found that apart from the skin, CC also originated in the mucosal membranes, esophagus, genitals, larynx, and oral mucosa. The first intraoral case was reported in 1977 by Flieger and Owiński [2]. Although approximately 80 cases of CC in the head and neck have been reported [3], cases of CC arising from the sinonasal mucosa are limited. A review of 77 cases of CC in the oral cavity found that two cases were detected in the maxilla, and both arose from the gingiva [3]. In addition, Janardhanan et al. suggested that more than one-third of the reported cases originated from the lower gingiva, the most common site in the oral cavity where CC has often been misdiagnosed initially [4]. These findings underline the need for a better understanding of the unique clinical and histopathological characteristics and biological behavior of this tumor [4].

Oroantral fistula (OAF) is an epithelialized pathological unnatural communication between the oral cavity and maxillary sinus. It can occur due to oral surgery, most commonly during the extraction of the upper molar and premolar teeth, and untreated larger defects often cause maxillary sinusitis. However, malignant transformation is rare.

Here, we describe a case of CC arising from long-standing (20 years) OAF and discuss the histopathological challenges associated with the diagnosis of CC involving the maxilla.

## Case presentation

**Figure 1:**
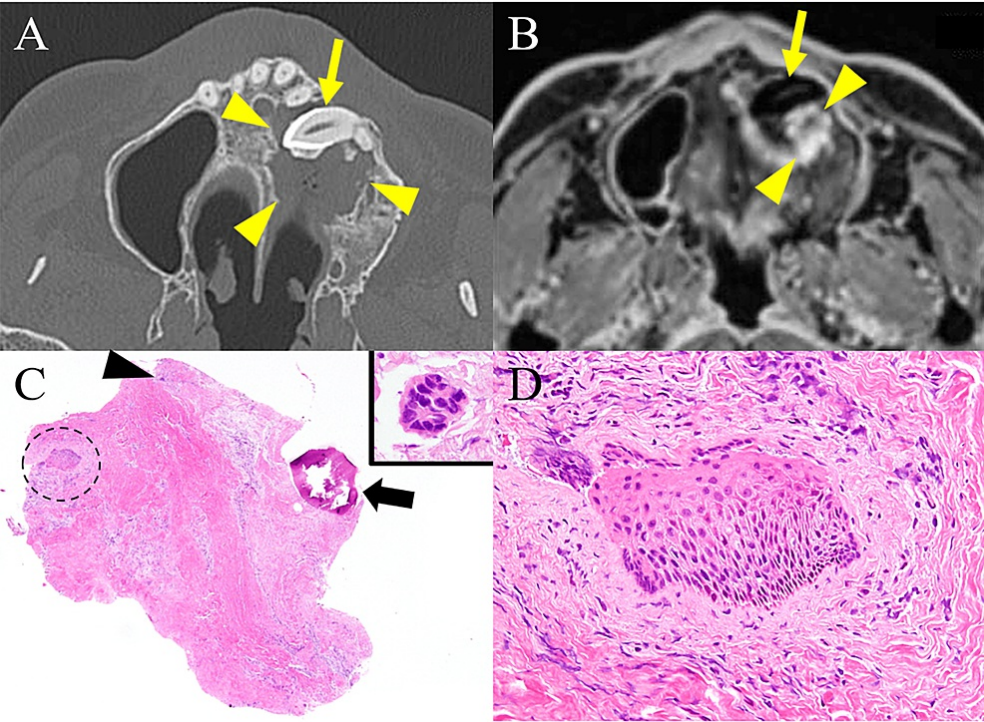
Images of the first admission and histopathology of the first biopsy CT revealed an osteolytic lesion (arrowheads) around the impacted tooth crown (arrow) extending from the left maxillary sinus to the left canine area (A). Contrast-enhanced T1-weighted modified Dixon MRI revealed a hyperintense lesion (arrowheads) with a central hypointensity, measuring 13 mm in diameter, in the distal cervical area of the impacted tooth crown (arrow) (B). A fragment of fibrous tissue, removed along with tooth extraction, contained a small dentinoid material component (arrow), a tiny odontogenic epithelial island (arrowhead) (inset), and a small piece of SE (dotted circle) (C). A high-power view of the SE revealed neither atypical changes, keratinization, nor basal cells palisading (D). CT: computed tomography; MRI: magnetic resonance imaging; SE: squamous epithelium

The patient was a 70-year-old Japanese man who had OAF following the extraction of a left upper canine tooth 20 years ago. He was a former smoker and had a history of hypertension and stroke. Four years ago, he was admitted to our hospital for the extraction of another impacted tooth in the left upper canine region. Computed tomography (CT) revealed an osteolytic lesion around the impacted tooth crown extending from the left maxillary sinus to the left canine area (Figure 1A). Contrast-enhanced T1-weighted modified Dixon magnetic resonance imaging (MRI) on the first admission revealed a hyperintense lesion with a central hypointensity, measuring 13 mm in diameter, in the distal cervical area of the impacted tooth crown (Figure 1B). The patient was diagnosed with postoperative change after maxillary sinusitis, and he underwent extraction of the impacted tooth with the surrounding tissue, followed by closing the OAF using a mucoperiosteal flap. A small specimen of fibrous tissue was submitted for histological examination. One-third of the specimen was composed of fibrous connective tissue with a small dentinoid fragment and an odontogenic epithelial island, which was reminiscent of the peri-coronal tissue or the dental follicle. The other part of the specimen showed significant fibrosis with mild lymphocytic infiltration and a small component of squamous epithelium (SE) (Figure 1C). This SE lacked keratinization and atypical changes. Basal cells were irregularly arranged without a palisading appearance (Figure 1D). The pathological diagnosis was suspicious of inflamed dentigerous cyst. Four months after surgery, the OAF recurred and was closed again using a mucoperiosteal flap. However, the fistula recurred soon after the second closure, and the patient was treated with denture closure and self-cleaning.

**Figure 2:**
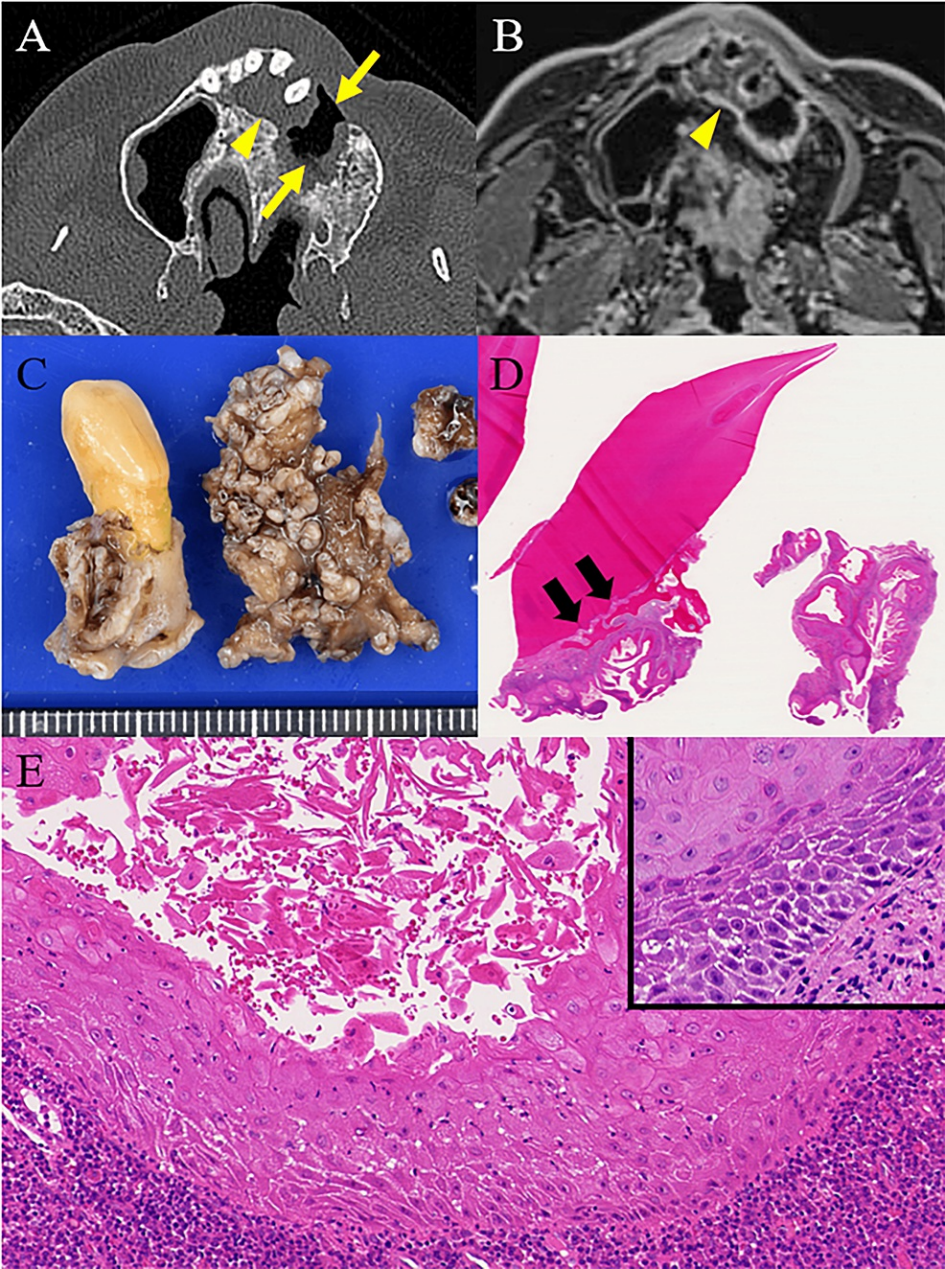
Images four years later and histopathology of the initial resection Four years later, CT revealed a 22-mm-sized radiolucent lesion (arrowhead) extending beyond the nasopalatine tract to the right side. It was close to the OAF (arrows) (A). Contrast-enhanced MRI also demonstrated a mass resulting in bone resorption in the maxilla (arrowhead) (B). Macroscopic examination of the resected specimen revealed that the periodontium of the incisor and the inner surface of the cystic wall had granular irregularities, such as a warty surface, but the mucosal surface was intact (C). In the low-power view, the tumor invaded the alveolar bone (arrows) (D). The epithelium was markedly hyperkeratotic and acanthotic with intraepithelial neutrophilic and lymphocytic infiltrates. Blunt spinous cells with conspicuous nuclei and small basal cells without palisading (inset) were noted (E). CT: computed tomography; OAF: oroantral fistula; MRI: magnetic resonance imaging

Four years after his first admission, CT revealed a 22-mm-sized radiolucent lesion extending beyond the nasopalatine tract to the right side (Figure 2A). Contrast-enhanced MRI also revealed a mass that resulted in maxillary bone resorption (Figure 2B). The mucosa of the upper gingiva was intact. The patient underwent surgical resection for the removal of the lesion and extraction of the incisors because the possibility of an odontogenic tumor could not be ruled out. The periodontium of the incisor and the inner surface of the cystic wall showed granular irregularities, such as a warty surface, but the mucosal surface was intact (Figure 2C). Histologically, the lesion was an irregularly shaped cystic endophytic papillary proliferation of SE covered by abundant keratinizing layers. The cystic lesion with an interconnected lumen, mimicking rabbit burrows, invaded the alveolar bone (Figure 2D). The proliferating SE was well-differentiated, markedly hyperkeratotic, and acanthotic. It was composed of small basal cells without palisading and large polygonal spinous cells with prominent eosinophilic cytoplasm but lacked a granular cell layer. Cytologic atypia was absent or minimal and limited to the basal and parabasal layers. Mitotic figures were rarely observed. Intraepithelial neutrophilic and lymphocytic infiltrates surrounding the epithelium were detected (Figure 2E).

**Figure 3:**
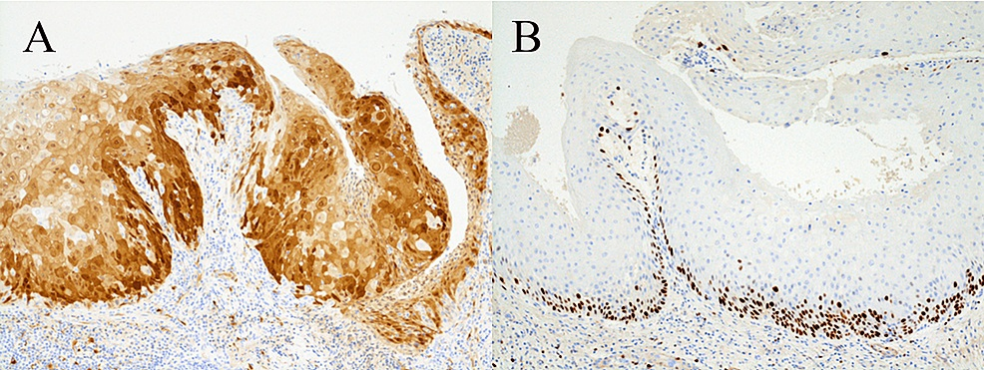
Immunohistochemical examination Strong and patchy nuclear and cytoplasmic staining with a p16 antibody was observed (A). Strong and nuclear positive cells for Ki67 were irregularly distributed not only in the para-basal layer but also in the basal layer (B).

Immunohistochemically the tumor cells showed strong and patchy nuclear and cytoplasmic reactions to p16 (E6H4, Roche Tissue Diagnostics, Basel, Switzerland) (Figure 3A). Strong and nuclear positive cells for Ki67 (MIB-1, DakoCytomation, Glostrup, Denmark) were irregularly distributed not only in the para-basal layer but also in the basal layer with scattered large positive nuclei (Figure 3B). The tumor cells were cytoplasmic positive for cytokeratin (CK)13 (KS-1A3, Leica Biosystems, Nussloch, Germany), and CK17 (E3, Leica Biosystems), but negative for CK19 (RCK108, DakoCytomation). A few basal cells were weakly positive for p53 (DO-7, DakoCytomation) (data not shown). Based on the above-mentioned pathological features, we diagnosed the lesion as CC.

**Figure 4:**
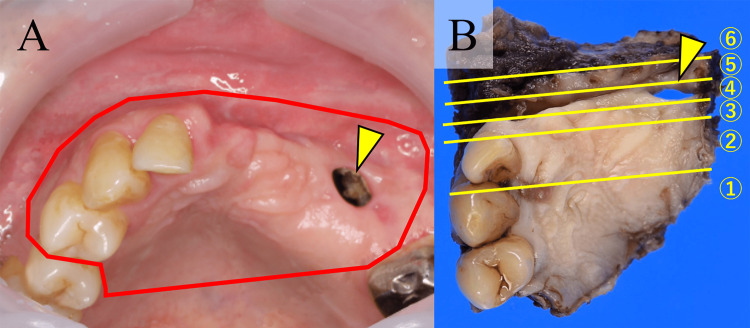
Oral view and macroscopic view of the additional resection The intraoral photograph showed the intact upper gingiva with an OAF (arrowhead) in the alveolar ridge. The resection range was shown by the red line (A). The specimen was sliced sagittally stepwise from the right to the left sides, as shown by the yellow lines. An arrowhead highlights the OAF (B). OAF: oroantral fistula

The mucosa of the upper gingiva was intact but an OAF remained in the left upper canine lesion. Therefore, in accordance with previous reports [5], complete surgical resection with a safety margin of > 10 mm was selected for the remaining CC and OAF (Figure 4A). Consequently, partial maxillectomy with the inferior turbinate and skin of the nasolabial folds was performed (Figure 4B).

**Figure 5:**
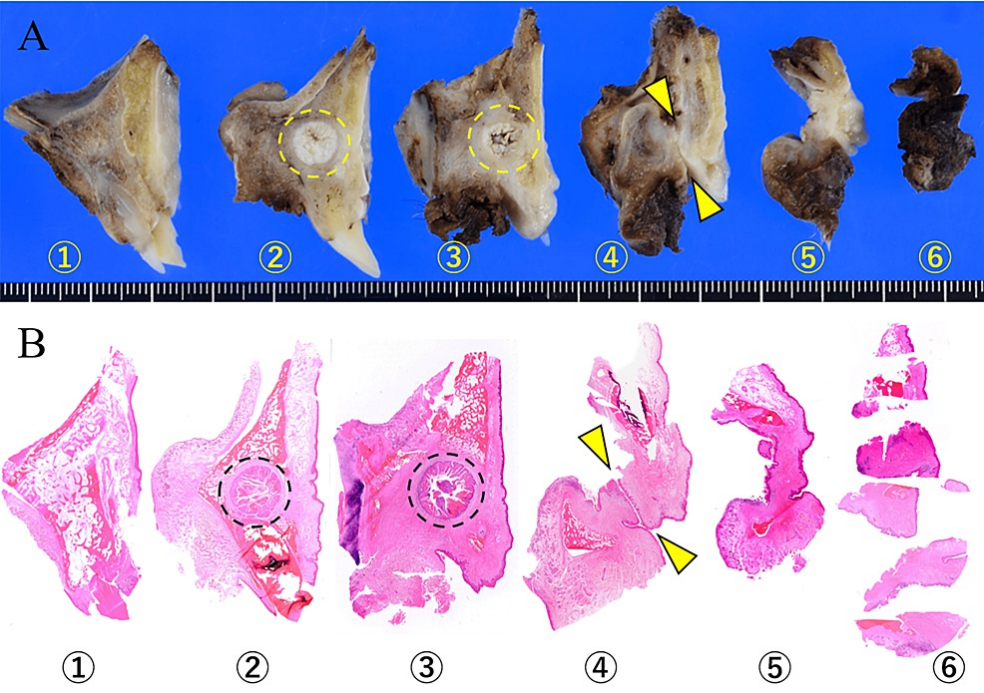
Sliced sections of the additional resection Cystic lesions (dotted circle) were serially found within the sliced sections numbered 2 and 3, and the OAF (arrowhead) was in section 4 (A, B). OAF: oroantral fistula

Macroscopically, cystic lesions remained within the main parts of the sliced specimens (Figure 5A, 5B).

**Figure 6:**
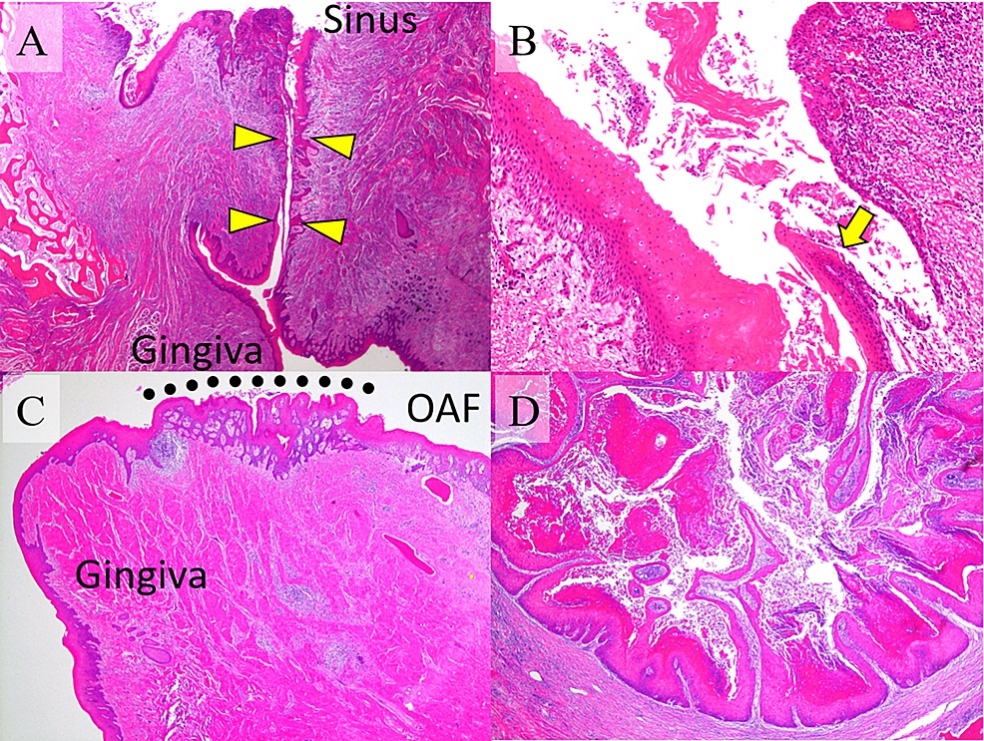
Histopathology of the additional resection The OAF (arrowheads), extending from the oral cavity to the maxillary sinus, was entirely covered by the SE (A). The sinus mucosa was partially replaced by atypical and hyperkeratinized SE. The arrow indicates a transition between the SE and respiratory columnar epithelium (B). The covering SE of the OAF partially exhibited CC with exophytic and endophytic papillary proliferation (dots) (C). The intraosseous lesion showed endophytic papillary proliferation of the SE with marked keratinization, similar to the findings observed in the initially excised specimen (D). OAF: oroantral fistula; SE: squamous epithelium; CC: carcinoma cuniculatum

Histologically, the OAF extending from the oral cavity to the maxillary sinus was entirely covered by the SE (Figure 6A). SE predominantly covered the fistula on the gingival side; however, an abrupt transition into the respiratory epithelium was noted on the sinus side (Figure 6B). The SE covering the OAF partially demonstrated exophytic and endophytic papillary proliferation without severe atypia, which was considered a part of the rest of CC (Figure 6C). The remaining intraosseous lesion showed endophytic papillary proliferation of the SE with marked keratinization, similar to the findings observed in the initially excised specimen (Figure 6D). Finally, we considered CC arising from OAF. Although the surgical margin was within 1 mm in parts, tumor cells were absent in all surgical margins. Strict follow-up was performed, and neither recurrence nor metastasis was detected after one year.

## Discussion

CC of the oral cavity is a rare variant of SCC. Preoperative diagnoses are often incorrect in almost all cases due to clinicians’ lack of awareness about this condition. Histologically, CC is difficult to diagnose, especially in the early stages, because of its mild atypia and well-differentiated nature. Initially, we could not make a correct diagnosis of the first biopsy specimen, which was diagnosed as “inflamed dentigerous cyst”. Retrospectively, a small fragment of SE found in the inflamed connective tissue was potentially a part of the carcinoma. CC is not only under-recognized among pathologists but is also susceptible to erroneous diagnosis, and it is often mistaken for other common benign and malignant pathological entities, such as reactive hyperplasia, verrucous carcinoma (VC), papillary SCC, conventional SCC, or odontogenic keratocyst (OKC). Sun et al. reported an incidence of 2.7% for CC after reviewing 540 cases of oral SCC [6]. However, because of numerous clinical and histologic similarities between this variant and the more widely described VC, the exact incidence of oral CC may have been underestimated. Notably, a comparative study of the clinical and pathological aspects between CC and VC of the oral mucosa has been performed, and the diagnostic criteria for differentiating the two entities have been defined [7,8]. CC shows a blunt papillary or cobblestone-like surface and has infiltrates with keratin plugging and local destruction; in contrast, VC exhibits predominantly exophytic growth with a papillary verrucous surface and a more restrained pushing border. Interestingly, cytological atypia is absent or mild in both CC and VC. However, other studies have documented diagnostic challenges between CC, VC, papillary SCC, and conventional SCC [3,9]. Compared with CC, papillary SCC and conventional SCC have more cytological atypia. Our case showed a cobblestone-like surface, endophytic growth with keratin-filled cysts, and mild to absent atypia. These findings match the features of CC. The prognosis of CC is worse than that of VC but better than that of both papillary SCC and conventional SCC. It is important to distinguish CC from other variants of SCC for prognosis and treatment, and it is more important to distinguish CC from benign lesions, such as hyperplasia, inflammation, and OKC. Invasion towards the intraosseous region suggests a malignant nature, but a reactive or benign lesion. The CC forms a cyst-like cavity with progression, which shows features of a keratinizing cyst and multiple epithelial islands. The parakeratinized epithelium lining the cyst and keratin-filled luminal space along with the epithelial islands in the stroma, which mimic daughter cysts, are the potential reasons for the misdiagnosis of OKC. Some discrimination points between OKC and CC have been discussed by Janardhanan et al. [4]. OKC arises within the bone, grows along the medullary cavity, and rarely invades the gingiva. Histologically, the epithelium is characterized by a scalloping parakeratotic surface and palisaded hyperchromatic basal cell layer composed of cuboidal to columnar cells. However, CC arises from the gingival or alveolar mucosa and invades the underlying bone. The wavy surface and palisading of basal cells are absent in the CC. In our case, the tumor originated from the OAF and invaded the maxilla and the alveolar bone. Morphologically, OKC shows characteristic features of epithelium, five to eight cell layers thick, without rete ridges. The basal layer is palisading with columnar or cuboidal cells [10]. However, the current lesion lacks such features, which allowed us to rule out OKC from the differential diagnosis.

We initially regarded this lesion as benign hyperkeratosis, OKC or VC. Based on the clinical and histological characteristics, a diagnosis of CC arising in the OAF was made, as discussed below. Retrospectively, the difficulty in closing the OAF suggests that the lesion was a tumor. The cystic lesion connected to the OAF and extending beyond the nasopalatine tract is not typical of OAF with osteomyelitis.

Chen et al. proposed a semiquantitative histologic scoring system for diagnosing CC in esophageal tumors; the presence of each of the following features is given 1 point: (1) hyperkeratosis, (2) acanthosis, (3) dyskeratosis, (4) deep keratinization, (5) intraepithelial neutrophils, (6) intraepithelial neutrophilic microabscess, (7) cytologic atypia, (8) koilocyte-like cells, and (9) keratin-filled cyst/burrows, and then total score is calculated [11]. On a scale of 0 to 9 points, a cutoff score of ≥7 has a specificity of 100% for CC in mucosal biopsies. This case met seven of the nine criteria, including cytological atypia in the basal cells, with the exceptions of intraepithelial neutrophilic microabscess and koilocyte-like cells (Table 1). According to the scoring system, the current case was estimated to have a score of 7, which is consistent with that of CC. In addition, Chen et al. compared esophageal biopsy specimens of CC with those of non-neoplastic lesions. Therefore, similar studies are needed for oral CC, especially those comparing it with reactive hyperplasia or conventional SCC, which is occasionally found in the oral cavity.

**Table 1:** Semiquantitative histologic scoring system for diagnosing carcinoma cuniculatum

Histologic scoring scale	This case	Score
Hyperkeratosis	present	1
Acanthosis	present	1
Dyskeratosis	present	1
Deep keratinization	present	1
Intraepithelial neutrophils	present	1
Intraepithelial neutrophilic microabscess	absent	0
Cytologic atypia	present	1
Koilocyte-like cells	absent	0
Keratin-filled cyst/burrows	present	1
	total score	7

Although Sun et al. found lower expression of Ki-67, p53, and p63 in CC than in SCC and VC [6], immunohistochemistry has limited diagnostic utility. Some cases have revealed that CC is negative for CK19, which is an odontogenic epithelial marker [4,12]. In contrast, OKC is usually positive for CK19. Immunohistochemical staining of p16 was performed in two studies; Goh et al. reported negative results in two cases [13], whereas Allon et al. demonstrated three positive cases out of five cases [12]. Our case showed a positive pattern (strong and patchy nuclear and cytoplasmic staining) similar to the positive cases in Allon et al.’s study. Although p16 has been considered a surrogate marker for high-risk human papillomavirus infection, human papillomavirus has not been identified in oral CC [12,14,15]. Finally, considering immunohistochemical findings, this case was diagnosed with CC, and CK19 negativity might exclude OKC.

Smoking, alcohol consumption, and trauma have been indicated as other causative factors; however, a clear etiology for oral CC is yet to be established. Our patient had a 20-year history of OAF and smoking; therefore, we suspected that chronic inflammation might have caused CC.

CC is locally aggressive and is associated with a low risk of distant metastasis. The long-term prognosis of the lesion appears to be favorable following appropriate surgical treatment with a safety margin. Nodal dissection is not necessary unless it is clinically indicated. However, the efficacy of chemotherapy and radiotherapy is controversial.

This case was suspected to be of OAF origin, but the evidence was imperfect, which might be a limitation of this study. Because the lesion was surgically removed twice, we were unable to directly assess the relationship between the primary portion of the CC and the OAF. Fortunately, the final resected specimen revealed the rest of the CC within the maxillary bone and the OAF, which could prove the OAF origin.

## Conclusions

To the best of our knowledge, this is the first well-documented case of CC arising from an OAF. Clinicopathological correlation and proper histopathological evaluation are critical to avoid the underdiagnosis of oral CC and its confusion with other tumors. Delays in diagnosis cause extensive involvement and other complications associated with the tumor. CC involving the maxillary sinus may require extended total maxillectomy, resulting in facial defects.

In conclusion, it is important to note that CC can arise from OAF. In cases of OAF with a long-standing clinical history and a destructive or radiolucent lesion in the maxilla, CC should be considered in the differential diagnosis.
